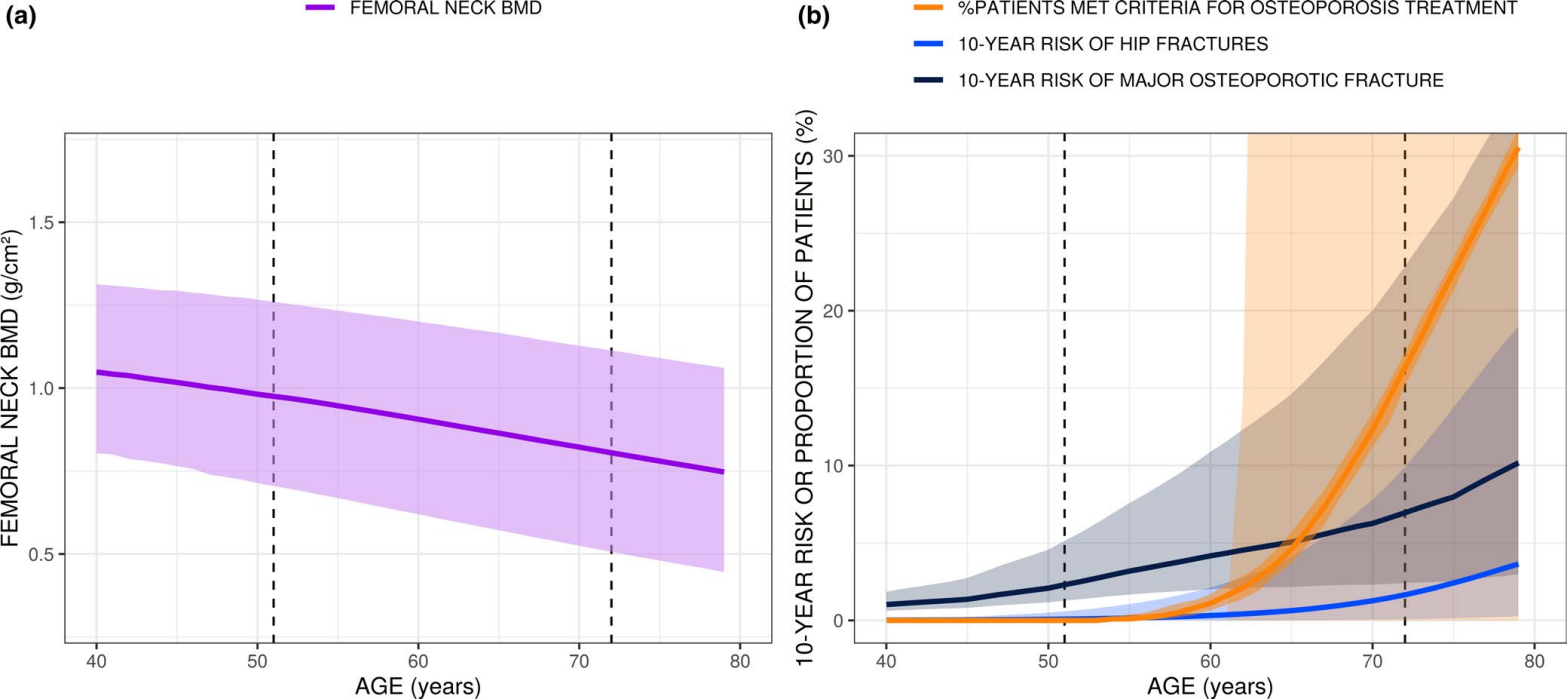# Corrigendum to: Integrating real‐world data and modeling to project changes in femoral neck bone mineral density and fracture risk in premenopausal women

**DOI:** 10.1111/cts.13188

**Published:** 2021-11-21

Denise Beck, Insa Winzenborg, Wei Gao, Nael M. Mostafa, Peter Noertersheuser, Stephanie E. Chiuve, Charlotte Owens and Mohamad Shebley, *Clin. Transl. Sci*., **14**. 1452‐1463. (2021) https://doi.org/10.1111/cts.13006

The article by Beck *et al*.,^1^ was published with an error in Figure 4b. The order and color assignments of labels in the published Figure 4b were inadvertently swapped in the original figure and do not correctly describe the data in Figure 4b. There are no changes to the data in this figure, only the color assignment has been corrected.

The corrected figure is shown below: